# Crystal layer growth with embedded carbon-based particles from effervescent tablet-based nanofluids

**DOI:** 10.1038/s41598-024-69605-y

**Published:** 2024-08-09

**Authors:** Naser Ali

**Affiliations:** Nanotechnology and Advanced Materials Program, Energy and Building Research Center, Kuwait Institute for Scientific Research, 13109 Safat, Kuwait

**Keywords:** Crystallization, MWCNTs, Suspension, Thermal conductivity, Dispersion stability, Effervescent agent, Materials science, Nanoscience and technology

## Abstract

Crystallization occurs as dissolved substances gradually solidify into crystal layers within a liquid, which can increase the capability of fluids to transfer heat. In this study, the growth of crystal layer in nanofluids produced from carbon-based effervescent tablets was examined. The tablets were fabricated by combining multi-walled carbon nanotubes (MWCNTs), sodium dodecyl sulfate (SDS), sodium phosphate monobasic (NaH_2_PO_4_), and sodium carbonate (Na_2_CO_3_). The effervescent tablets were formulated with MWCNTs, NaH_2_PO_4_, and Na_2_CO_3_ at a weight ratio of 1:5.1:2.26, respectively. These tablets were then immersed in distilled water (DW) and seawater (SW) to produce 0.05 vol.% to 0.15 vol.% MWCNT suspensions. Then, the dispersion stability, thermal conductivity, and crystal layer growth of the nanofluids were characterized. The results showed that the DW-based nanofluids were more stable than their SW-based counterparts. Additionally, the 0.05 vol.% DW-based suspension exhibited greater long-term stability than those of the 0.15 vol.% suspensions, whereas the SW-based nanofluid exhibited the opposite behaviour. The greatest increases in thermal conductivity were 3.29% and 3.13% for 0.15 vol.% MWCNTs in DW and SW, respectively. The crystallization process occurred in nanofluids that contained more than 0.05 vol.% MWCNTs and exhibited a greater growth rate in SW-based suspensions with high effervescent agent concentrations.

## Introduction

**Figure 1:**
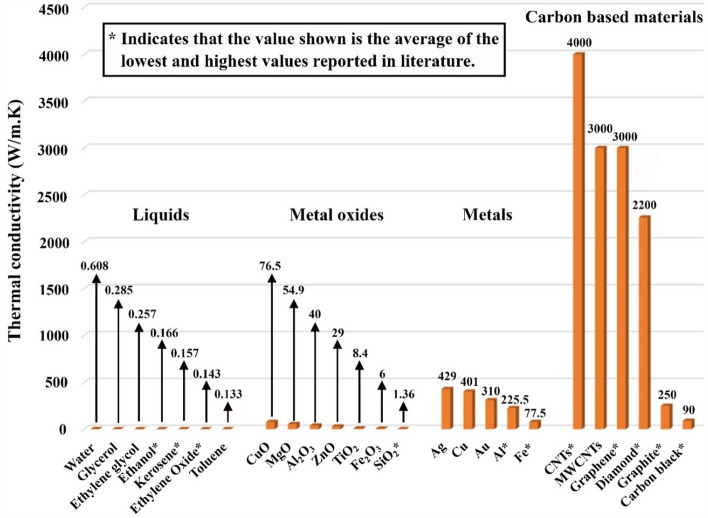
Thermal conductivity of particles and basefluids commonly used for producing suspensions^22^.

Since the 1900s, researchers have dedicated significant efforts to developing working fluids with physical and thermal properties beyond the standards of those conventionally used in scientific and industrial fields. Furthermore, the aggregation of solid particles ranging from mm to µm in size was first developed in ground-breaking work by Ahuja^1,2^ in 1975, Liu et al.^3^ in 1988, and researchers at Argonne National Laboratory (ANL)^4–6^ in 1992, who built upon the theoretical work of Maxwell^7^. These dispersions exhibit remarkably increased thermal properties compared with those of base fluids, which results from the substantially improved thermal conductivity (TC) of the aggregated particles compared to that of the host liquid. Figure 1 illustrates notable variations in TC between solid particles and liquids, focusing on feedstocks commonly used at 25 °C and 1 bar for fabricating suspensions^8–12^. Later, ‘nanofluids’ were introduced by Masuda et al.^13^ and then defined by Choi and Eastman^14^ in 1993 and 1995, respectively. In general, these suspensions are classified as advanced fluids that are formed by minimal concentrations of particles (ideally ≤ 1 vol. %), which are uniformly aggregated and smaller than ~ 100 nm in size, in non-dissolving base fluids^15^. Thus, when these particles are dispersed within a base fluid (e.g., water), which is used in most thermal applications as a working fluid, these dispersed nanomaterials can help modify the liquid thermophysical and optical properties^16^. The application of nanoparticles to increase thermal conductivity for various applications has been widely noted in the literature^17–19^. Recently, these advanced fluids have been successfully adopted in many research activities to improve the thermal performance of heat exchange devices that operate with liquids (e.g., solar collectors, computer coolant systems, and air conditioning lubricant systems)^20^. In general, two main approaches are used to produce these advanced types of suspensions^21^. The first route is defined as the single-step (or one-step) approach, and the second technique is called the two-step approach. In the single-step method, nanoparticles are formed and dispersed within the hosting liquid in a single stage. This approach exhibits several advantages, including the following: (1) the suspension exhibits a high physical dispersion stability, and (2) the method eliminates the need to handle and transport dry powders, as well as the need for spacing allocation^20^. However, this production approach is always associated with residues that are challenging to eliminate due to incomplete reactions and can only be employed to synthesize certain combinations of nanoparticles and liquids. In addition, the equipment needed for the one-step production approach is usually very complicated and expensive. In contrast, the two-step approach utilizes pre-prepared powders, which are then added to a base fluid of non-dissolving characteristics and dispersed through a mixing machine, such as a magnetic stirrer, homogenizer, or ultrasonicator. This approach exhibits several advantages, as any type of nanofluid can be produced, the method can be easily performed by individuals with decent skills, commercial powders are widely available worldwide, and the method can be adopted for small-scale as well as large-scale production. Due to these advantages, this production approach has always been favourable by many scholars working in this area of research. Nevertheless, mixing devices are a key factor in the suspension production process that uses a two-step approach; thus, applying this fabrication method in remote areas with limited electrical sources is challenging. Furthermore, suspensions created via this method (i.e., the two-step approach) exhibit less physical dispersion stability than those created via the one-step method. However, this limitation can be improved by including surfactants with the mixture at the fabrication stage or by performing surface functionalization with the particles before mixing them with the liquid.

Although these advanced suspensions have led to significant accomplishments in the field of thermal fluids, some challenges still exist, and it is important to overcome these challenges before introducing these colloidal materials to the industry. For instance, the process of preparing nanofluids is known to substantially impact the thermophysical properties and physical stability of the fluid^12,23^. Correctly executing the production process is crucial, as an inadequately executed process increases the likelihood of producing an unstable suspension. Consequently, the thermal properties of the suspension would gradually degrade over time due to particle separation from the carrier liquid. The relationship between nanofluid TC and dispersion stability has been demonstrated in the literature^20^. Scholars have concluded that the optimum TC can only be achieved once the particles are uniformly distributed within the base fluid (i.e., the suspension is physically stable), and vice versa. However, the drawbacks in terms of physical stability are mainly associated with the use of nanofluids under stationary conditions; thus, if the system remains under dynamic flow conditions, the physical stability of the dispersed particles should be less affected. Furthermore, the cost-effectiveness of the nanomaterial feedstock, along with its potential impact on the environment and human health, must be addressed for acceptance in industry.

**Figure 2:**
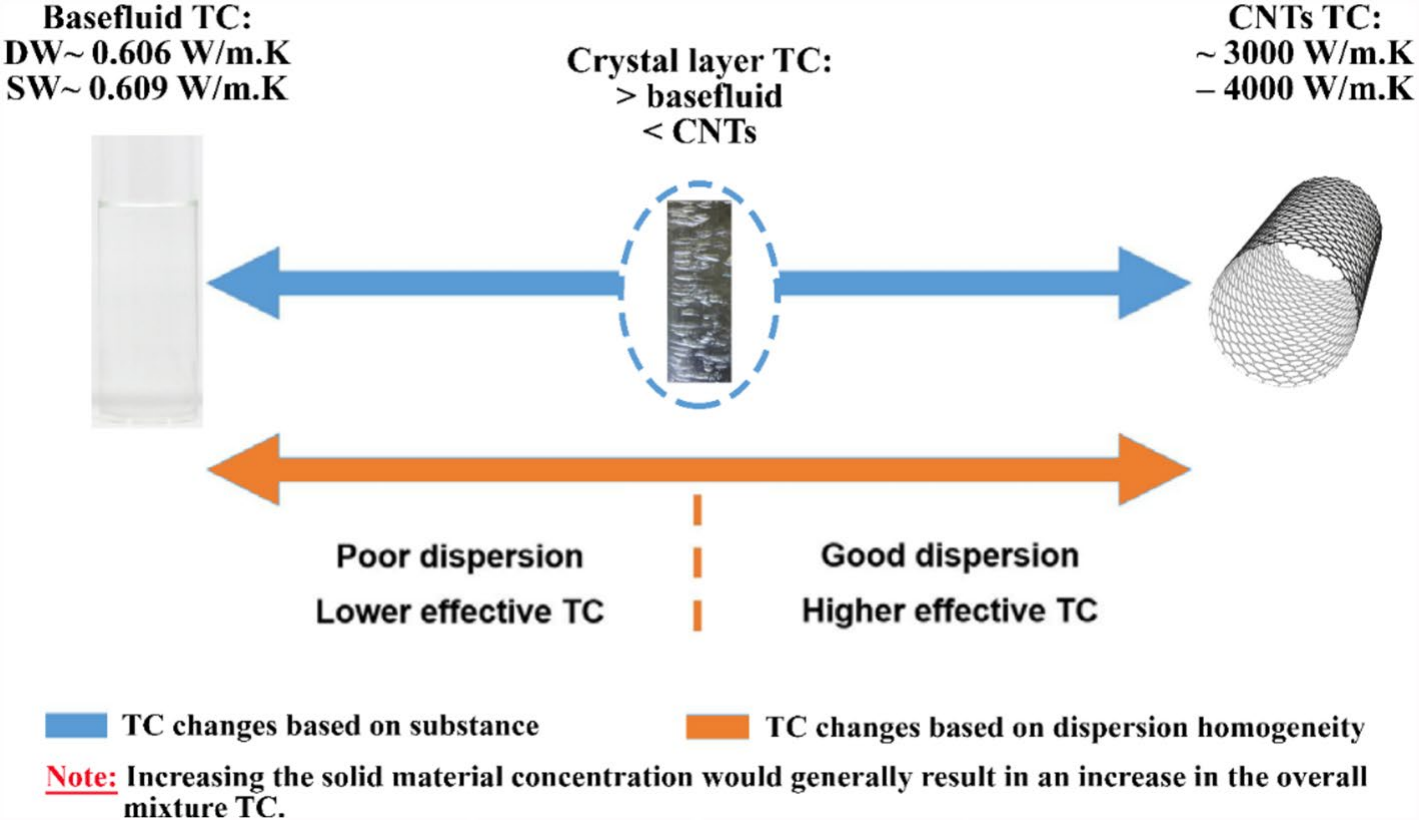
Variation in the thermal conductivity of different substances and dispersion stability conditions, in which DW, SW, and CNTs represent distilled water, seawater, and carbon nanotubes, respectively.

In contrast, a new nanofluid production approach was recently introduced by Ali et al.^21^ and Alsayegh et al.^24^ to fabricate dispersions for thermal applications. Scholars have integrated effervescent tablet technology with suspension science to fabricate carbon-based nanofluids for thermal application. Moreover, they demonstrated that their approach could generate cost-effective suspensions with improved thermal properties compared to those produced conventionally. In general, effervescent tablets are produced by combining a mixture of solid powders made from bicarbonate (or carbonate) alkaline salts (e.g., sodium bicarbonate, sodium carbonate, potassium bicarbonate, or potassium carbonate) and organic acids (e.g., adipic, citric, fumaric, tartaric acid, or malic)^25^. These tablets tend to react chemically once immersed in a polar liquid (e.g., water), which causes them to dissolve gradually while releasing bubbles containing carbon dioxide (CO_2_) gas. The generated bubbles then tend to provide a sufficient buoyant force for mixing the included sold particles in the base fluid. In addition to CO_2_, gases such as hydrogen and oxygen can be generated via effervescent tablet technology^26,27^. However, the right effervescent agent must be selected, and one (or more) active material(s) must be present. Thus, to fabricate effervescent tablet-based nanofluids, researchers would simply add the effervescent tablet containing nanoparticles in its matrix to a base fluid and wait for the chemical reaction and generation of CO_2_ bubbles. These bubbles provide the necessary buoyant force to fully disperse the particles within the hosting liquid and hence complete the nanofluid production within a few minutes^28^. Therefore, no physical devices are required, and only minimal experience is needed for researchers to obtain these suspensions. Hence, when the effervescent tablet technological approach is utilized, the obstacles that limited the old processes in remote areas or places without electrical sources are eliminated. Since the effervescent agents included in the effervescent tablets contain acidic and alkaline salts, they tend to form crystal layers of distinctive geometric shapes over time. The formation of crystal layers is governed by the principles of crystallization, a process in which molecules or ions in a solution assemble in an organized manner to form a solid crystal lattice^29^. The introduction of chemicals into the solution plays a crucial role in initiating this process by providing the necessary conditions for the nucleation and growth of crystals^30^. These crystal layers possess different characteristics, such as transparency, cleavage, anisotropy, and thermal properties. They can grow in various shapes and sizes, depending on the type of chemical used, their concentration within a solution, and the environmental conditions involved in their growth. Thus, when crystal layers are present in a nanofluid, they are expected to improve the suspension TC, as shown in Fig. 2.

Inspired by above background, this research investigated crystal growth in effervescent tablet-based nanofluids as well as their thermal properties, which has never been reported in literature to the best of the author’s knowledge, demonstrating the novelty of this work. First, effervescent tablets were fabricated by combining multi-walled carbon nanotubes (MWCNTs), sodium phosphate monobasic (NaH_2_PO_4_), sodium dodecyl sulfate (SDS), and sodium carbonate (Na_2_CO_3_) at different concentrations. Next, the as-fabricated effervescent tablets were immersed in DW and SW to produce carbon-based nanofluids ranging from 0.05 vol.% to 0.15 vol.%. Following the production phase, the dispersion physical stability, crystal growth time, and suspension TC were determined. The results are expected to help researchers improve the performance of thermal applications, such as heat exchangers and solar still desalination systems.

## Experimental procedure

### Materials and consumables

The commercial nanomaterials, effervescent agents, and surfactant powders used were MWCNTs, NaH_2_PO_4_ (acidic salt), Na_2_CO_3_ (alkali salt), and SDS (anionic), respectively. The specifications of the powders used are listed in Table 1.

**Table 1:** Specification of the as-purchased commercial powders.

Powder type	Category	Purity (wt.%)	Other technical info	Supplier
MWCNTs	Nanomaterial	95	Inner diameter (ID): 5–10 nm Outer diameter (OD): 30–50 nm Axial length: 5–15 µm	SkySpring Nanomaterials Inc
NaH_2_PO_4_	Effervescent agent	≥ 99.0	ReagentPlus^®^	SIGMA-ALDRICH Inc
Na_2_CO_3_	Effervescent agent	≥ 95.5	ReagentPlus^®^	SIGMA-ALDRICH Inc
SDS	Surfactant	≥ 98.5	ReagentPlus^®^	SIGMA-ALDRICH Inc

In addition, transparent glass vials (height: 9.5 cm, OD: 4 cm, and thickness: 0.32 cm) were supplied by Glass Solutions Ltd., which were later used for hosting the suspensions. The DW was produced from a ZIQ7000T0C DW supply unit (Merck Millipore Co.), whereas the SW was collected from the State of Kuwait Sea (coordinates: 29° North and 47° East). The specifications and total dissolved solids (TDS) of the previous two liquids are tabulated in Supplementary Table 1S.

### Feed powder characterization

X-ray diffraction (XRD) analysis was used to determine the elemental content of the commercial powders. A CuK_α_ X-ray source at a 2θ diffraction angle was used in the XRD device (SmartLab, Rigaku Co.) along with a working power of 9 kW. The scanning rate used was 1°/min, whereas the diffraction scanning angle range and incidence beam employed were 15°–75° and 0.1°, respectively. The previous was done to determine the Bragg peaks in each sample, which were used to reveal and confirm the elemental content. The particle morphology was explored using field emission scanning electron microscopy (FE-SEM) (JSM-7800F, JEOL Co.), and the level of powder purity was determined through an energy dispersive X-ray spectroscopy (EDS) unit that was integrated with the FE-SEM device. In contrast to carbon-based nanomaterials, effervescent agents and SDS powders were initially coated with a thin layer of gold to improve their electrical conductivity^22^. In addition, the employed working distance was 10 mm, and the accelerating voltage used was between 8 and 10 kV (depending on the sample type). Since the density of the MWCNTs (${\rho }_{MWCNTs}$) must be measured to determine the volume percentage (vol. %) of MWCNTs before they are added to the basefluid, this value was measured at room temperature using a gas pycnometer–volumetric system (Model 1, HumiPyc TM.) and was found to be ~ 2.097 g/cm^3^.

### Effervescent tablets fabrication and nanofluid production

**Figure 3:**
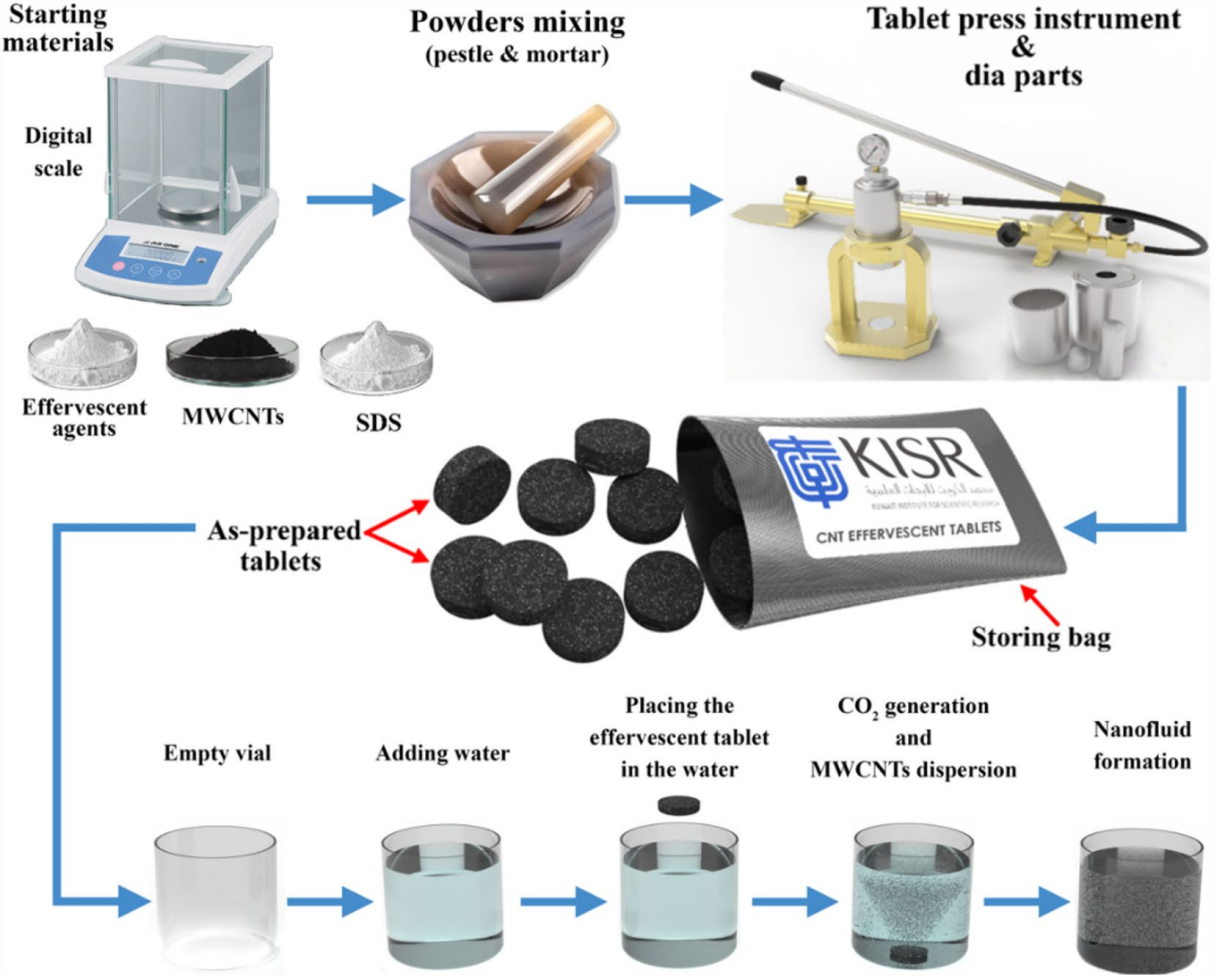
Illustration of effervescent tablet fabrication and the suspension production processes.

Initially, effervescent tablets were formed and used to produce nanofluid samples. This was achieved by mixing the nanomaterial with the surfactant, i.e., MWCNTs and SDS, at a 1:1 weight ratio. A second mixing prosses was then conducted to the as-prepared MWCNTs-SDS powder after adding the effervescent agents (i.e., NaH_2_PO_4_, and Na_2_CO_3_) at a wight ratio of 1:5.1:2.26. Both mixing steps were conducted via hand mixing using a mortar and pestle tool. Additionally, the weight ratio between the carbon material and surfactant was selected based on the work of Almurtaji et al.^31^ and Ali^32^, as the ratio leads to good physical stability in the suspension. For the weight ratio between the MWCNTs and the effervescent agents, the selection approach is explained in detail in the Supplementary Material and has been shown to be effective in forming well-dispersed carbon-based nanofluids. Following the two-stage mixing, the powder was consolidated at 100 kN using a tablet press instrument and 25 mm ID die to fabricate the effervescent tablets. Next, the as-fabricated tablets were immersed in two types of liquids, namely, DW and SW, to form suspensions ranging from 0.05 to 0.15 vol.%. The vol. % was calculated based on 90 mL of base fluid and using the mixing theory^12,33^ as follows:

1 $$vol. \%= \frac{{V}_{MWCNTs} }{{V}_{bf}+ {V}_{MWCNTs} } \times 100;$$

2 $$vol. \%= \frac{\frac{{m}_{MWCNTs} }{{\rho }_{MWCNTs}} }{{V}_{bf} + \frac{{m}_{MWCNTs} }{{\rho }_{MWCNTs}} } \times 100;$$

where ${V}_{MWCNTs}$, ${V}_{bf}$, and ${m}_{MWCNTs}$ are the volume of the MWCNTs (mm^3^), volume of the base fluid (mL), and mass of the MWCNTs (mg), respectively. Notably, the base fluid temperature was 25 °C, and the nanofluid fabrication process was complete once the immersed tablet was fully dissolved. The previous effervescent tablet fabrication and suspension production processes are illustrated in Fig. 3.

### Nanofluid characteristics

#### Stability analysis

Three analyses were conducted to determine the stability of the as-prepared suspension, namely, an image capturing approach, UV‒Vis spectroscopy, and zeta potential^34^. In the first method, images of the samples were taken after initial preparation and after 20 days. On the other hand, UV‒Vis analysis was conducted with a SHIMADZU Co. UV-2600 system at wavelengths ranging from 200 to 800 nm and with a 1.0 nm measuring interval. The zeta potential of the suspensions was measured using a Malvern Instruments Co. Nano Series—Zetasizer after 0.75 mL was placed in a DTS 1061 folded capillary cell, which was obtained from the same supplier.

#### Thermal properties

The TC of the as-prepared suspensions was measured after the fabrication process. This was performed by measuring the thermal properties three times, with 10 min between each measurement, and then averaging the three obtained values. The previous experiments were performed using a transient hot-wire system of type THW-L2, which was supplied by Thermtest Instruments, Inc., and operates based on an ASTM D7896-19 standard. Furthermore, the device had an error rate of less than 5%, according to the manufacturer. A thermoelectric dry bath was used to maintain the sample temperature at 25 °C throughout the measurement process.

#### Crystallization growth

The crystal layer growth in the as-prepared suspensions was determined by analysing the captured images of the samples. This includes the starting time needed for the layer to grow and up to the duration for complete formation of the crystals. Additionally, a Lab compound microscope of type SM201 (Ikarus Technology Co.) was used to determine whether any traces of the MWCNTs were captured within the crystal layers. Notably, the temperature and pressure at which the crystal layers formed were 25 °C and 1 atm, respectively.

## Results and discussion

### X-ray diffraction analysis

**Figure 4:**
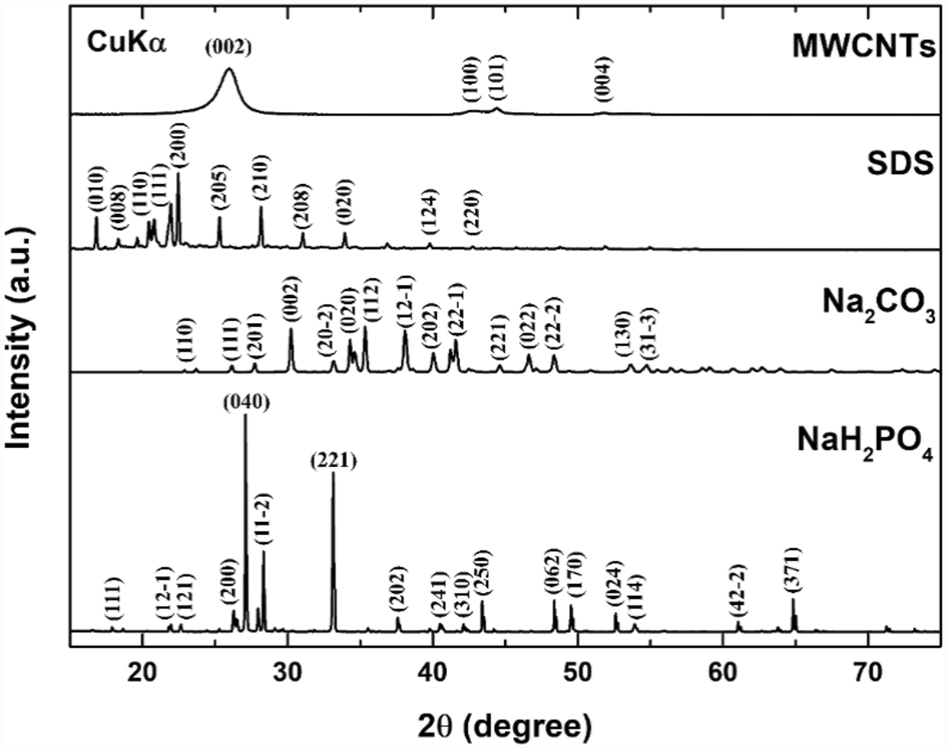
X-ray diffraction analysis of MWCNTs, SDS, Na_2_CO_3_, and NaH_2_PO_4_ powders.

The generated XRD diffraction patterns of the as-received powders, MWCNTs, SDS, Na_2_CO_3_, and NaH_2_PO_4_ are shown in Fig. 4. Furthermore, the Bragg peaks in the generated results for the MWCNT, SDS, Na_2_CO_3_, and NaH_2_PO_4_ as-received samples were found to correspond to the system databases with PDF numbers 00-058-1638, 00-039-1996, 05-001-0004, and 01-084-0112, respectively. The generated patterns were also consistent with other available data in literature^35–38^.

The Scherrer formula was used to determine the crystallite size as follows^39^:

3 $${D}_{hkl}= \frac{F\lambda }{{\beta }_{hkl}cos{\theta }_{hkl}}$$

The parameters in Eq. (3) correspond to a shape factor ($F$) of 0.90, a CuK_α_ wavelength ($\lambda$) of ~ 0.154 nm, a complete width of 0.5 for the maximum of the diffraction peak of $hkl$ (${\beta }_{hkl}$), and a peak $hkl$ Bragg angle (${\theta }_{hkl}$). Moreover, the corresponding $hkl$ coordinates for the highest peaks for the examined powders and the average crystallite size are listed in Table 2.

**Table 2:** XRD analysis of the as-received powders, including the highest $hkl$ peak and average crystallite size.

Material type	Highest $hkl$ peak	Avg. crystallite size (nm)
MWCNTs	0 0 2	8.3
SDS	2 0 0	47.7
Na_2_CO_3_	1 1 2	83.9
NaH_2_PO_4_	0 4 0	33.9

### FE-SEM and EDS characterization

**Figure 5:**
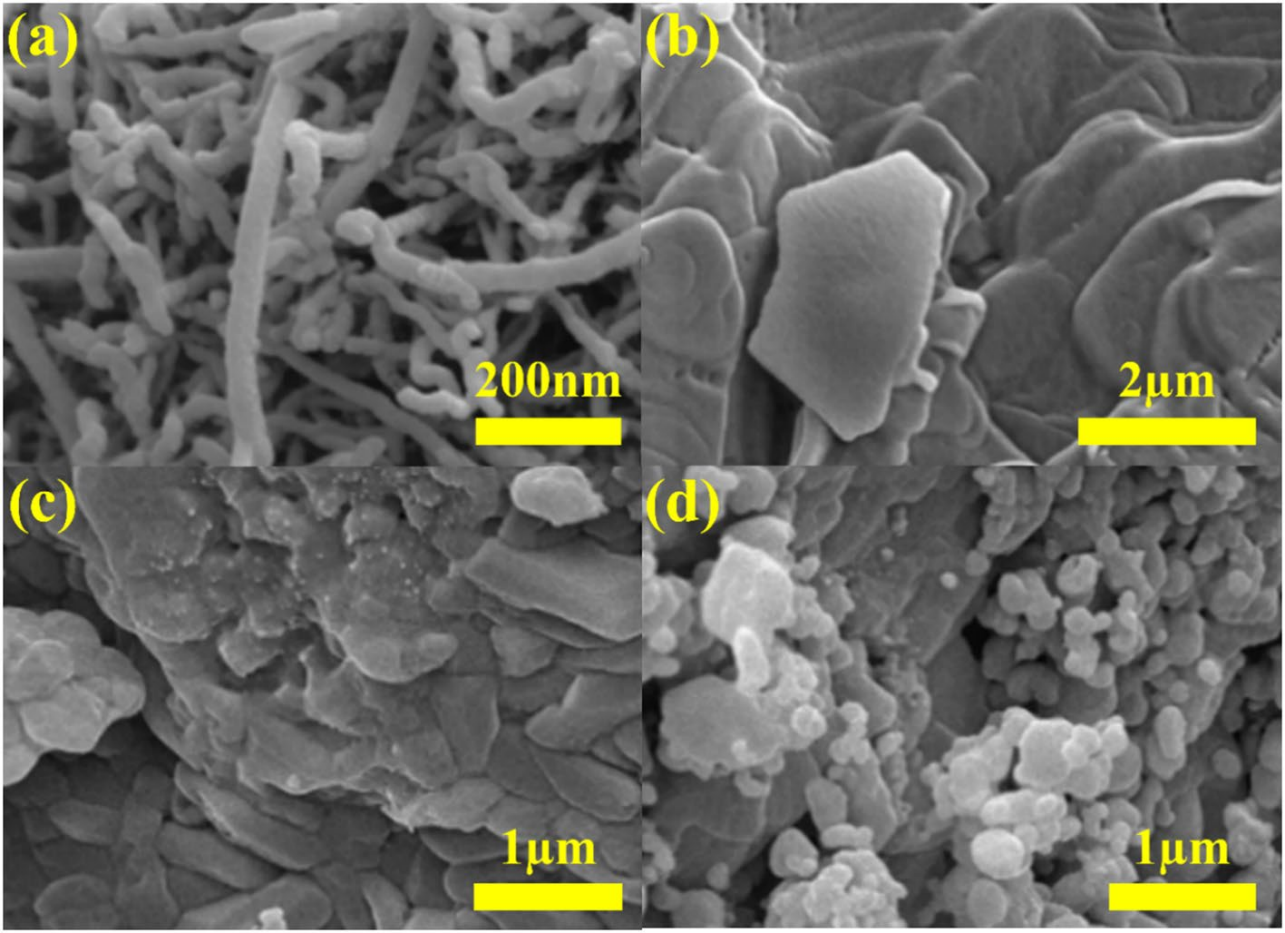
Morphological FE-SEM images of the samples: (**a**–**d**) MWCNTs, SDS, Na_2_CO_3_, and NaH_2_PO_4_, respectively.

The visual morphological images of the as-received nanomaterial, solid surfactant, and effervescent agent powders are shown in Fig. 5a–d. Figure 5a shows that the shape of the examined material is tube-like, which corresponds to the structure of carbon nanotubes (CNTs)^40^. The tube-like materials can also be seen clustered with each other, which can be linked to the high surface area-to-volume ratio of the carbon-based material^41^. Furthermore, the examined CNTs exhibited an OD in the range of 23–49 nm. As shown in Fig. 5b,c, the structure of the materials are non-uniform but exhibits a flake-like morphology. The diameter of the structures shown in Fig. 5b was between 0.4 and 1.8 µm, whereas the range in Fig. 5c was between 0.2 and 1.2 µm. According to Fig. 5d, the particle shapes are generally closest to a sphere. The range in the examined material structure is between 0.08 and 0.6 µm. Notably, the images shown in Fig. 5a–d represent the as-received powders of MWCNTs, SDS, Na_2_CO_3_, and NaH_2_PO_4_, respectively.

**Figure 6:**
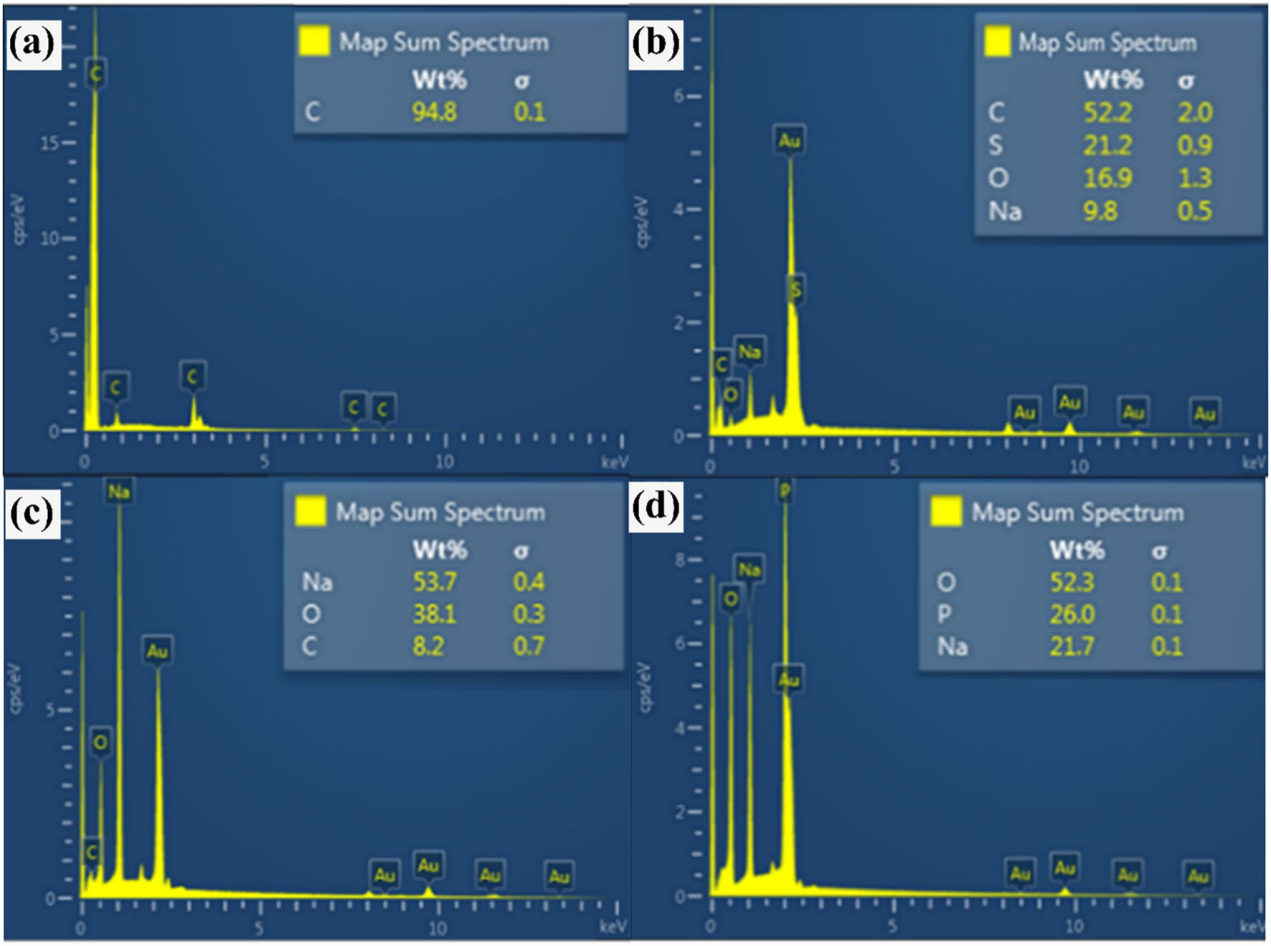
Elemental content of the samples examined via EDS spectroscopy, in which (**a**–**d**) show the MWCNTs, SDS, Na_2_CO_3_, and NaH_2_PO_4_, respectively.

The EDS analyses of the as-obtained powders are illustrated in Fig. 6a–d. From the EDS spectra, it was found that the wt.% of the element contained in Fig. 6a is 94.8 carbon; Fig. 6b is 52.2 carbon, 21.2 sulfur, 16.9 oxygen, and 9.8 sodium; Fig. 6c is 53.7 sodium, 38.1 oxygen, and 8.2 carbon; and Fig. 6d is 52.3 oxygen, 26 phosphorus, and 21.7 sodium. Thus, the results of Fig. 6a–d clearly represent the main elements of MWCNTs, SDS (i.e., NaC_12_H_25_SO_4_), Na_2_CO_3_, and NaH_2_PO_4_, respectively. In general, both the FE-SEM and EDS analyses correspond well with the manufacturer’s descriptions.

### Physical stability of the suspension

**Figure 7:**
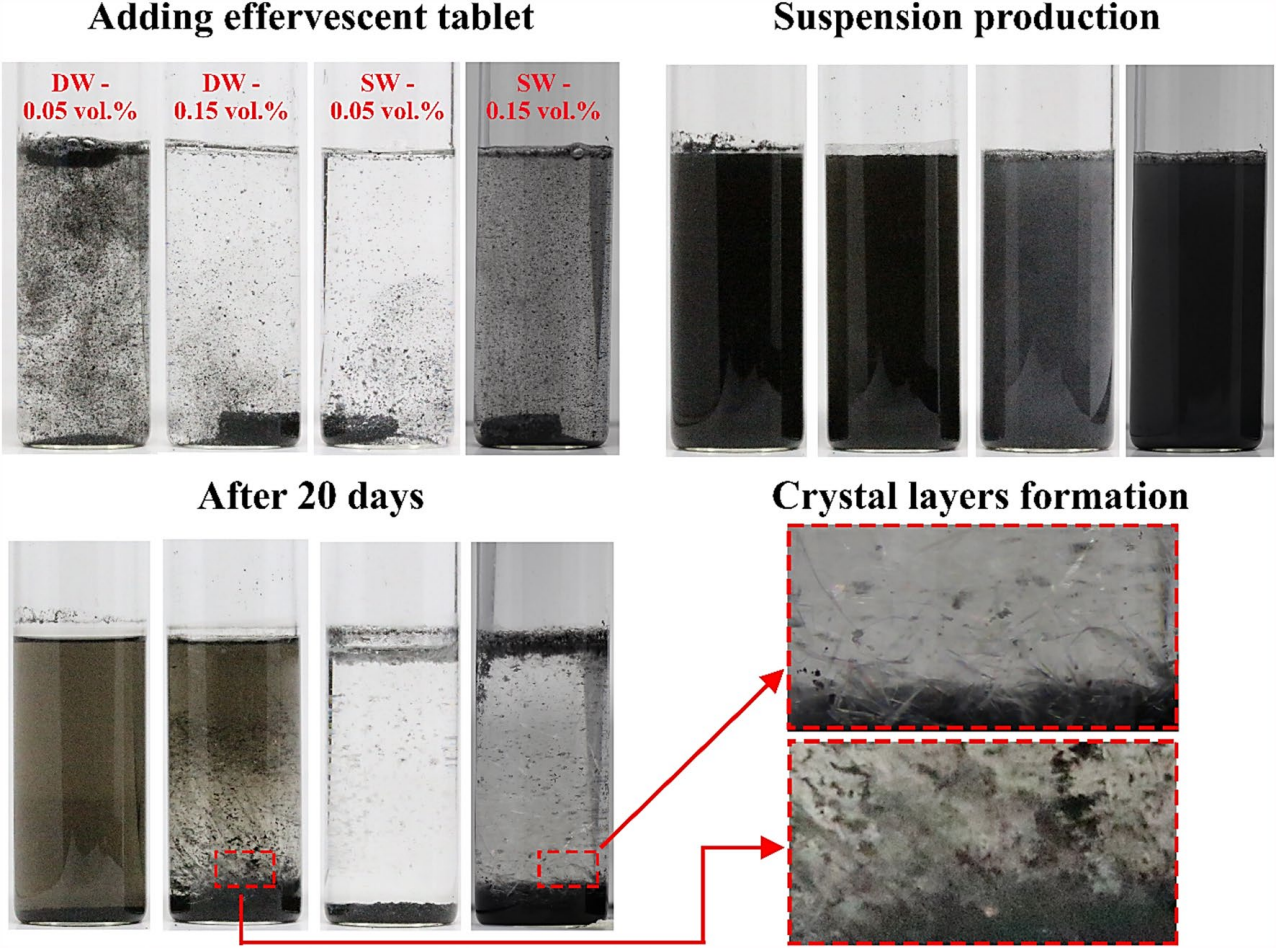
Sedimentation photographical showing the stability tests performed for suspensions fabricated with DW and SW.

The image-capturing stability evaluation approach was employed for the as-prepared suspensions and after 20 days, and the results are shown in Fig. 7, which revealed the main findings. The first finding is related to the dispersion stability associated with the nanomaterial concentration and type of base fluid. Specifically, increasing the vol. % of MWCNTs in DW rapidly decreased the stability of DW over time. In contrast, the opposite behaviour was observed for SW-based suspensions, as the settling rate of the dispersed carbon-based material decreased with increasing MWCNT concentration. In addition, crystal-like layers were observed with increasing vol. % of the dispersed MWCNTs following a certain time for both types of base fluids, as illustrated in Fig. 7.

**Figure 8:**
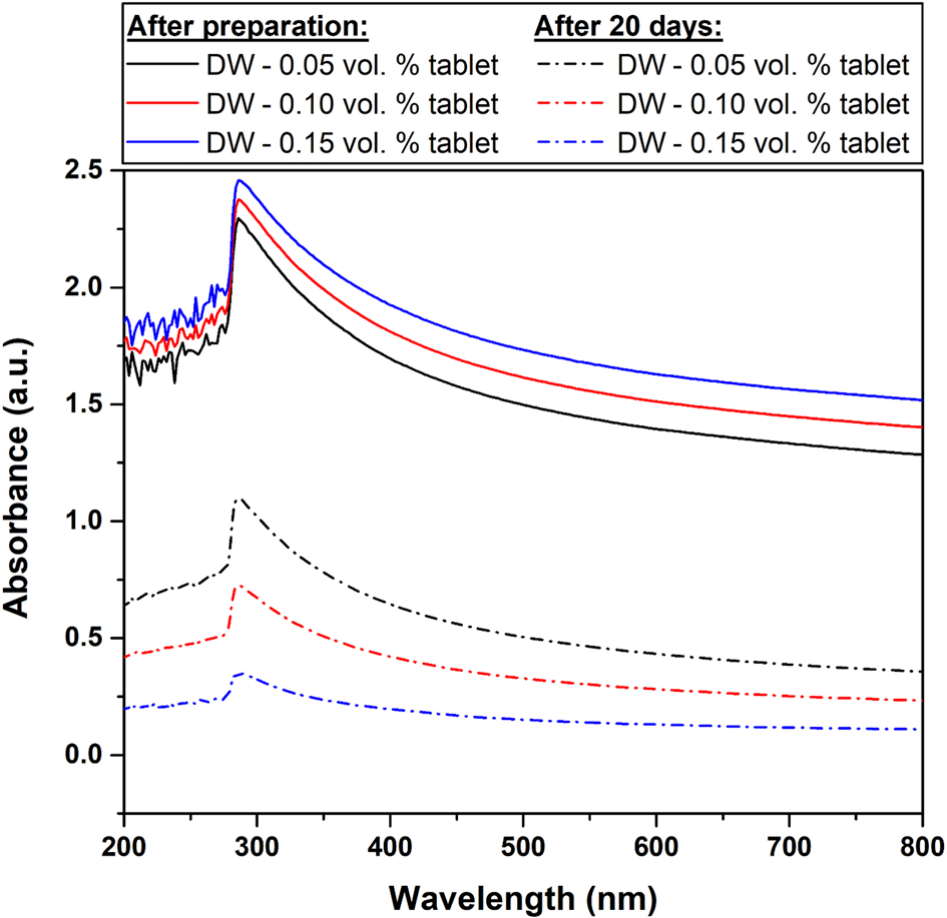
UV‒Vis analysis of the as-prepared DW-based suspensions directly after preparation and after 20 days.

**Figure 9:**
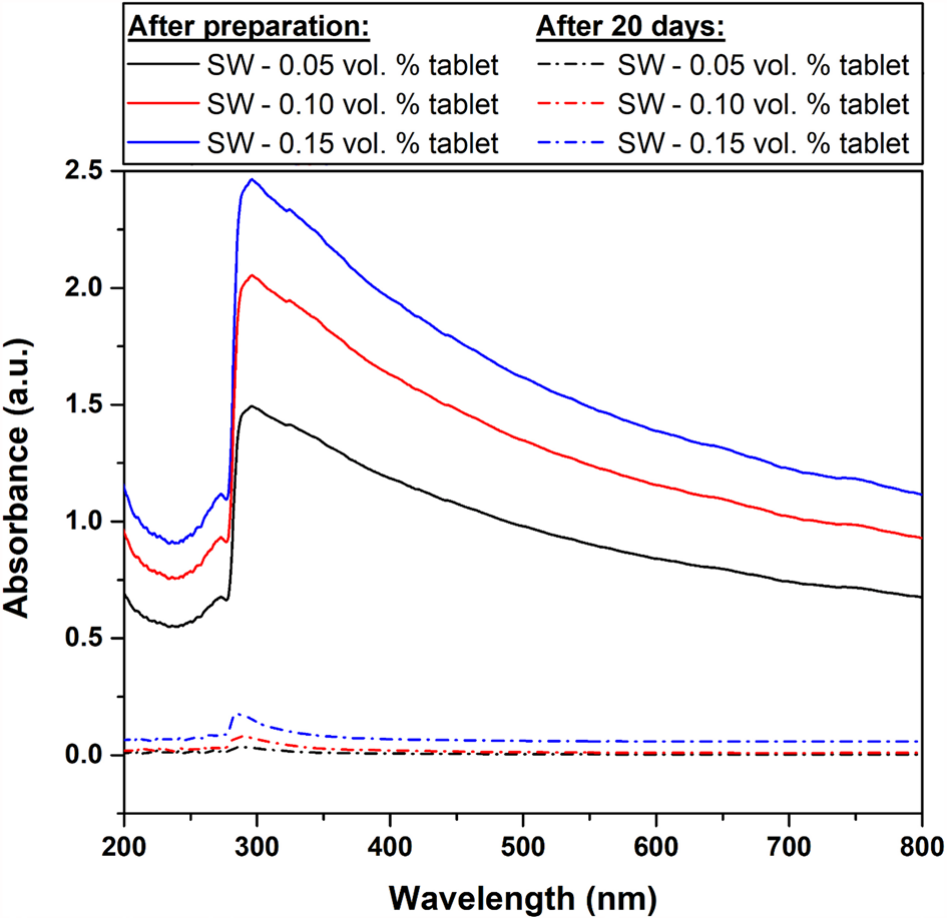
UV‒Vis analysis of the as-prepared SW-based suspensions directly after preparation and after 20 days.

**Figure 10:**
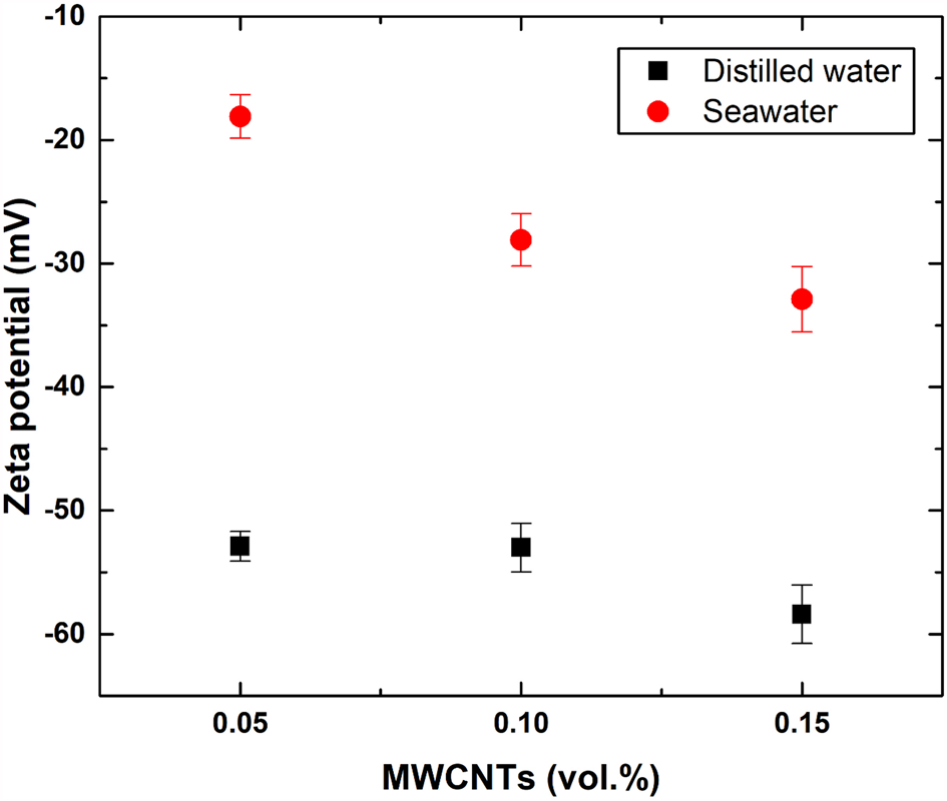
Zeta potential measurements of the as-prepared DW-based and SW-based nanofluids at different MWCNT concentrations.

For further stability clarification, UV‒Vis spectroscopy analysis was conducted on the as-fabricated DW-based and SW-based nanofluids, as demonstrated in Figs. 8 and 9. The UV‒Vis results confirm the findings of previous methods used to evaluate the image capture stability. However, the high level of TDS in the SW has shown a significant influence on the physical stability of the dispersed MWCNTs. Nevertheless, this effect is noticeably reduced when increasing the dispersed MWCNT concentration, as shown in Fig. 9. Both types of suspensions (i.e., DW-based and SW-based) were compared, and the DW-based nanofluids were found more stable than those fabricated with SW. Additionally, the SW-based dispersions almost fully clustered into forming sediments that separated from the hosting liquid with time. Furthermore, zeta potential measurements provided further insight into the stability of the as-prepared samples. For instance, Fig. 10 shows that DW-based suspensions are generally more stable than their SW-based counterparts. Moreover, the values obtained for the DW-based nanofluids (i.e., 0.05 vol. % to 0.15 vol. %) are in the − 52.9 mV to − 58.4 mV range, indicating good stability (i.e., between − 45 mV to − 60 mV)^34^. However, the 0.05 vol. % SW-based sample showed low stability (i.e., between − 15 mV to − 30 mV), whereas the 0.10 vol. % and 0.15 vol. % samples both demonstrated moderate stability (i.e., between − 30 mV to − 45 mV)^34^.

### Dispersion thermal properties

**Figure 11:**
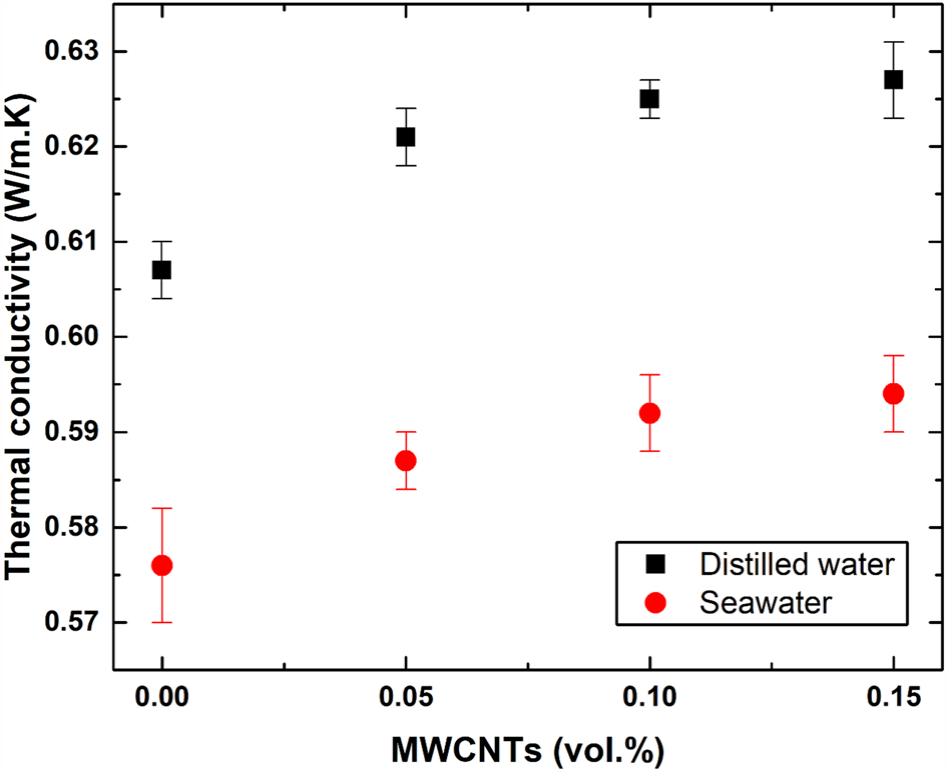
Thermal conductivity of the as-prepared DW-based and SW-based nanofluids at different MWCNT concentrations.

The measured TC of the as-fabricated suspensions is presented in Fig. 11. Furthermore, the thermal properties of the DW-based nanofluids were generally greater than their SW-based suspensions counterparts at similar MWCNT concentrations. The differences in TC between the SW-based nanofluids and DW-based nanofluids were found to be ~ 5.63% and ~ 5.41% for 0.05 vol. % and 0.15 vol. %, respectively. Regarding the increase in the thermal properties caused by the dispersed nanomaterials in the base fluid, the addition of 0.15 vol. % MWCNTs increased the TC of DW and SW by 3.29% and 3.13%, respectively. Additionally, the results in Fig. 11 revealed that the initial properties of the base fluid used was the primary factor influencing the thermal properties of both as-prepared nanofluids; the secondary factor was the dispersed nanomaterial concentration because it was relatively low. Nevertheless, other factors, such as dispersion stability, usually have a large impact on the TC of the suspension when the nanomaterials start to cluster into sediments^42^. However, this is rarely observed for freshly prepared suspensions.

### Crystal layer formation

**Figure 12:**
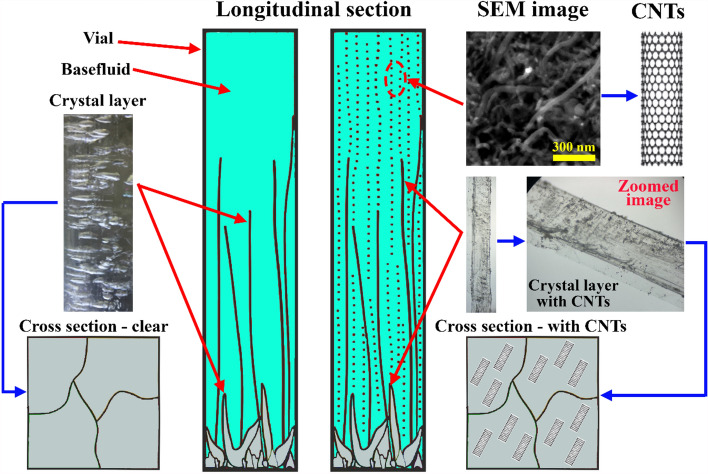
Crystal layer formation in aqueous liquids showing the final form of the crystal material once dispersed particles exist in the solution (right) and the crystal layer formed in a nanomaterial-free solution (left).

Crystallization or crystal layer formation in a liquid is influenced by the presence of the total dissolved solids in the base fluid and the effervescent agents that are included in the fabricated effervescent tablets. However, a certain duration of time and a constant temperature are needed for these layers to start growing in a host liquid^43,44^. Additionally, if crystal layers start to form in a suspension, they tend to capture some of the dispersed nanomaterial within them and on their outer surface. Figure 12 shows the formation of crystal layers in aqueous liquids from the effervescent agents with and without dispersed MWCNTs. The microscopy images of the crystal layer formed from the effervescent agents for both fluid causes (i.e., with and without dispersed nanomaterials) clearly show the presence of the MWCNTs embedded and on the surface of the crystal layer formed within the suspension, as shown in Fig. 12.

**Figure 13:**
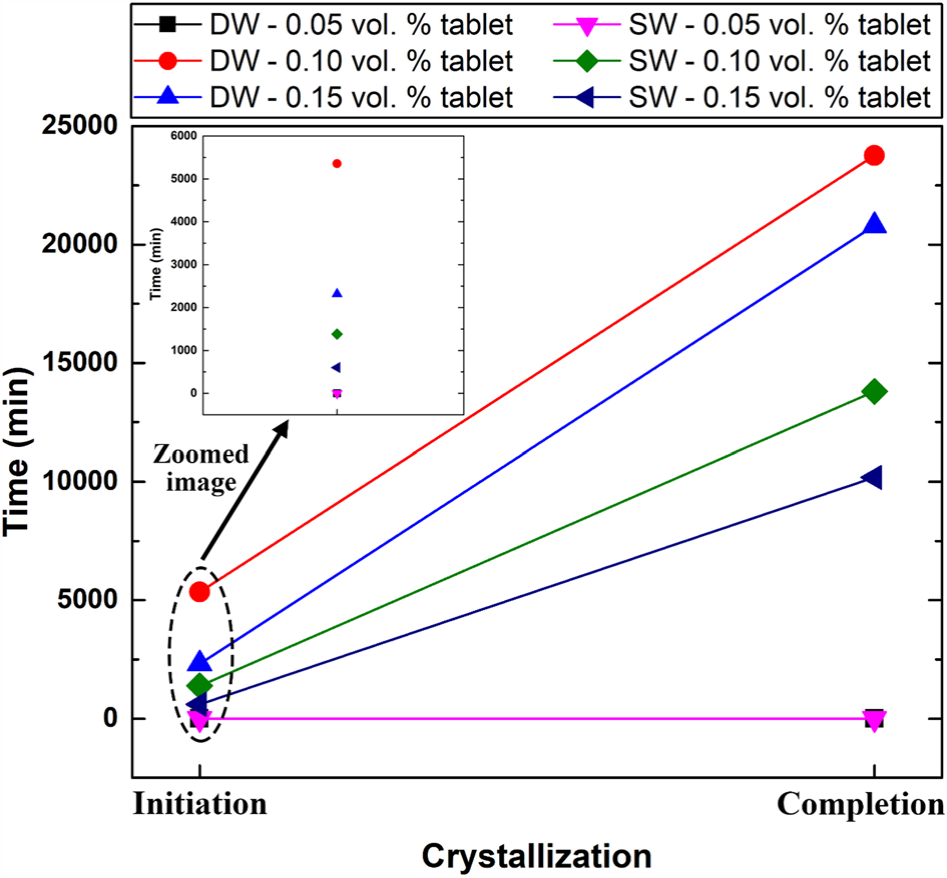
Time necessary for the crystal layers to start and complete their growth process for different effervescent tablet volumes and base fluid types. The 0.05 vol. % samples did not show any crystallization.

As mentioned earlier, the crystal layers start to grow after the effervescent agents are added to the liquid and pass through a stationary stage. Thus, the initiation of crystallization growth and the time needed for their complete formation were determined, as illustrated in Fig. 13. As shown in Fig. 13, neither the DW-based nor the SW-based suspensions fabricated with 0.05 vol. % showed any crystallization, indicating that the amount of effervescent agents included in the effervescent table along with the base fluid TDS was not sufficient to initiate the crystallization process. However, the effervescent tablets containing more vol. % (i.e., 0.10 vol. % and 0.15 vol. %) of nanomaterial all developed crystal layers because the MWCNT content is related to the amount of effervescent agents in the effervescent tablet via the weight ratio, as mentioned in "Effervescent tablets fabrication and nanofluid production". Therefore, increasing the vol. % of MWCNT would increase the amount of effervescent agent in the effervescent tablet, and vice versa. Furthermore, the starting time necessary for the crystal layers to initiate and complete their growth was less in the SW-based nanofluids than in the DW-based suspensions. For instance, the crystallization process in the SW-based nanofluid of 0.10 vol. % and 0.15 vol. % was found to be 1382 min (initial)–13,791 min (complete) and 602 min (initial)–10,181 min (complete), respectively. On the other hand, the DW-based nanofluid with 0.10 vol. % and 0.15 vol. % required a duration of 5354 min (initial)–23,756 min (complete) and 2317 min (initial)–20,795 min (complete), respectively. This result confirms that the presence of dissolved solids in the base fluid plays a major role in the crystallization process, as increasing the dissolved solid content in the base fluid increases the growth rate of the crystal layers, and vice versa.

## Conclusion

In this study, the growth of crystal layers in MWCNTs-based nanofluids formed by dissolving effervescent tablets in DW and SW was investigated, as well as their thermal properties and dispersion stability. The effervescent tablets were fabricated by combining a mixture of MWCNTs, SDS, NaH_2_PO_4_, and Na_2_CO_3_. A 1:5.1:2.26 weight ratio of nanomaterial with surfactant, NaH_2_PO_4_, and Na_2_CO_3_ was selected for the tablet formulation. The tablets were then used to produce DW-based and SW-based nanofluids with MWCNT concentrations ranging from 0.05 to 0.15 vol. %. Afterwards, the as-prepared suspensions were characterized in terms of their dispersion stability, thermal conductivity, and crystal layer growth. The dispersion stability results indicated that DW-based nanofluids are more stable than SW-based suspensions. Additionally, increasing the vol.% of MWCNTs reduced and increased the long-term stability (i.e., 20 days) of the DW-based and SW-based nanofluids, respectively. On the other hand, the DW-based dispersions were generally found to exhibit higher thermal conductivity than that of the SW-based suspensions. Furthermore, the greatest increase in the base fluid thermal properties was achieved with 0.15 vol. %, with values of 3.29% and 3.13% for DW and SW, respectively. The primary factor that influenced the thermal conductivity was the type of base fluid, while a secondary effect was observed for the concentration of MWCNTs. In terms of crystal layer growth, a minimum effervescent agent concentration is needed to initiate the growth mechanism. Additionally, base fluids containing pre-dissolved solids, such as SW, help increase the crystallization process. The time needed for crystal layer formation in the DW-based and SW-based suspensions of 0.15 vol. % was 2317 min (initial)–20,795 min (complete) and 602 min (initial)–10,181 min (complete), respectively. These findings can be used for future solar still desalination applications, in which the formed crystal layers containing the nanomaterials can be used to increase the thermal conductivity of the working fluid and potentially increase the solar absorption. However, further research is needed to control the crystallization rate and dispersion stability in SW before progressing towards industrial applications can be achieved.

### Supplementary Information

Supplementary Information.

## Data Availability

The datasets used and/or analysed during the current study available from the corresponding author on reasonable request.
