# Supplementary material for: Crystal layer growth with embedded carbon-based particles from effervescent tablet-based nanofluids
Source: Sci Rep. 2024 Aug 9;14:18554. doi: 10.1038/s41598-024-69605-y (PMC11315990; doi:10.1038/s41598-024-69605-y)
Supplement: Supplementary file 1 — Supplementary Information. [file 41598_2024_69605_MOESM1_ESM.docx]

**Supplementary Material**

Table 1S shows the specifications of the distilled water and seawater that were used as basefluids in the conducted work.

Table 1S. Distilled water and seawater specification.

| **Analysis type** | **Unit** | **DW** | **SW** |
| --- | --- | --- | --- |
| Appearance | **–** | Colourless | Colourless |
| Conductivity | mS/cm | <0.001 | 20.4 |
| Thermal conductivity | W/m.K | 0.606 | 0.576 |
| Viscosity | mPa.s | 0.89 | 1.19 |
| Density | g/cm^3^ | 0.983 | 1.010 |
| Specific heat capacity | kJ/kg.K | 4.182 | 4.009 |
| pH | **–** | 5.77 | 7.26 |
| Bi-carbonate alkalinity | mg/L | 2.86 | 121.84 |
| Carbonate alkalinity | mg/L | 0 | 0 |
| OH-alkalinity | mg/L | 0 | 0 |
| Total alkalinity | mg/L | 2.86 | 121.84 |
| Sodium | mg/L | <0.01 | 4,443.50 |
| Calcium | mg/L | <0.01 | 931.26 |
| Magnesium | mg/L | <0.01 | 668.9 |
| Potassium | mg/L | <0.01 | 108.46 |
| Strontium | mg/L | <0.01 | 13.05 |
| Barium | mg/L | 0.15 | 0.03 |
| Iron | mg/L | <0.01 | 0.04 |
| Lithium | mg/L | <0.01 | 0.1 |
| Silicon | mg/L | <0.01 | 10.56 |
| Boron | mg/L | <0.01 | 3.3 |
| Chloride | mg/L | <0.01 | 7,128.12 |
| Sulfate | mg/L | <1 | 3,400 |
| TDS (calculated) | mg/L | 3.02 | 16,829.16 |

The following shows the approach used in determining the mass ratio between $NaH_{2}PO_{4}$ and $Na_{2}CO_{3}$.

**Chemical reaction**

The chemical reaction between $NaH_{2}PO_{4}$ and $Na_{2}CO_{3}$ is as following:

$NaH_{2}PO_{4}+Na_{2}CO_{3}\to Na_{2}HPO_{4}+NaHCO_{3}$ (1)

$NaH_{2}PO_{4}+NaHCO_{3}\to Na_{2}HPO_{4}+CO_{2}+H_{2}O$ (2)

**Conversion**

For total conversion between $NaH_{2}PO_{4}$ and $Na_{2}CO_{3}$, a stoichiometric ratio of 2:1is required. Meaning that 2 moles of $NaH_{2}PO_{4}$ should be included with each mole of $Na_{2}CO_{3}$. The weight of each substance is as following:

1 mole of $Na_{2}CO_{3}$ = 105.988 g

2 moles of $NaH_{2}PO_{4}$ = 239.954 g

1 mole of $CO_{2}$ = 44.01 g

Thus, the reaction between 1 mole of $Na_{2}CO_{3}$ and 2 moles of $NaH_{2}PO_{4}$ will generate 132.03 g of $CO_{2}$.

**Mass ratio**

The mass ratio between the outcome of 239.954 g of $NaH_{2}PO_{4}$ and 105.988 g of $Na_{2}CO_{3}$ 2.26:1. Considering the generated amount of $CO_{2}$ needed to physically disperse the particles, a weight ratio of 1:5.1:2.26 was used for the nanomaterial and surfactant, $NaH_{2}PO_{4}$, and $Na_{2}CO_{3}$, respectively. Notably, lower mass ratios could not experimentally provide sufficient buoyant force to fully disperse the nanomaterial within the basefluid.
